# The use of strip-seeding for management of two late-season invasive plants

**DOI:** 10.1016/j.heliyon.2019.e01772

**Published:** 2019-05-22

**Authors:** Amanda Dechen Silva, Leslie M. Roche, Elise S. Gornish

**Affiliations:** a Universidade de São Paulo, Brazil, Rua da Reitoria 109, Cidade Universitaria São Paulo, 05508-900 Brazil; b University of California, Davis. One Shields Avenue, Davis, CA 95616, USA; c University of Arizona, 1064 East Lowell Street, Tucson, AZ 85721, USA

**Keywords:** Agriculture, Environmental science, Lactuca serriola, Restoration, Convolvulus arvensis

## Abstract

The spread and persistence of weedy plants in rangelands highlight the need for refinement of existing management techniques and development of novel strategies to address invasions. Strip-seeding – the strategic seeding of a portion of an invaded area to reduce costs and enhance success – is an underutilized management approach that holds promise for reducing weed dominance in grassland habitats. A strip-seeding experiment was established in 2011 in a California grassland where portions (between 0-100%) of invaded plots were seeded with native grasses. In 2016, we assessed the height, above-ground biomass and flower production of two late-season invasive plants: field bindweed and prickly lettuce. We found significant reductions in plant height and flower production (for both target invasives), and biomass (for field bindweed) in many of the seeded strips compared to the unseeded strips. Smaller seed applications demonstrated similar or better utility for weed control compared to greater seed applications, suggesting that this approach can be effective while reducing labor and materials cost of typical restoration management approaches. We did not find evidence that seeded strips provided invasion resistance to unseeded strips. This is possibly due to the lag in native species dispersal and establishment into contiguous unseeded strips, and suggests that strip-seeding might not provide invasion resistance to unseeded strips on timescales that are relevant to managers. However, this work does suggest that strip-seeding native species that overlap in phenology with target invasives can reduce late-season weed dominance on rangelands.

## 1 Introduction

Invasive plants are a major challenge for the maintenance of productive rangelands. The presence of invasive plants can reduce land value by inhibiting biodiversity, depressing forage productivity, and depleting soil and water resources (Duncan et al., 2004). However, typical invasive species management approaches such as prescribed fire, herbicide application, mowing and targeted grazing might not be compatible with current rangeland practices or they might demonstrate limited utility in significantly reducing invasive plant cover and reproduction (e.g., Davy et al., 2015; James et al., 2015). Feasible, cost-effective weed management strategies must be identified through science-based methods to facilitate widespread, successful control of invasive plants by land owners and land managers in rangeland systems (e.g. Gornish and James, 2016).

Incorporating approaches from the field of ecological restoration into weed management might provide utility for controlling nonnative species. Although uncommon (Kettenring and Adams, 2011), seeding or planting desired plants in invaded areas after an initial weed control treatment (e.g. herbicide) holds promise for managing invasive plants. This is because reseeding can reduce existing weed establishment (O'Dell et al., 2007) while also increasing plant community resistance to future invasion (Funk et al., 2008). This can occur for two reasons. First, seeding species that are functionally similar in resource use to target weeds increases the magnitude of competition experienced by invasive species (Connell, 1983). Second, seeded or planted species can take up space in bare patches created by conventional weed control methods. These patches are often disproportionately invaded due to an absence of competition (e.g. Shumway, 1995).

Spatially patterned seeding with desirable species is a component of vegetation management that might be particularly suitable for weed control. Spatially patterned seeding, or strip-seeding, refers to a restoration approach where seed is strategically applied to a portion of a total degraded area in order to enhance coverage of desired plant species (Rayburn and Laca, 2013). This approach is potentially more cost-effective than traditional restoration methods because smaller areas are treated, which reduces costs associated with labor and plant materials. This technique is also compatible with, and may improve, forage production, suggesting that this approach can be especially useful in working landscapes, such as rangelands. Finally, spatially patterned seeding approaches can result in greater restoration success than traditional restoration methods because areas that are most likely to facilitate seed germination and emergence (e.g. sites that retain moisture or are protected from wind) can be targeted for seeding. Despite the potential value of strip-seeding as a tool for invasive species management, formal research that identifies best management strategies for this approach is surprisingly uncommon (James et al., 2015; but see Blumenthal et al., 2003).

We tested the capacity of using native grass species in a spatially patterned seeding design to reduce biomass and reproduction of two late season invasive plants: field bindweed (*Convolvulus arvensis* L.) and prickly lettuce (*Lactuca serriola* L.). These invasive plant species are two of the most important weeds across agricultural systems worldwide (Holm et al., 1991; D'Andrea et al., 2009). Field bindweed, a vining perennial broadleaf with extensive roots is native to Europe and Asia, reproduces primarily through vegetative growth (Liebman et al., 2001). Prickly lettuce, a Mediterranean winter or summer annual or biennial upright broadleaf is drought and disturbance tolerant and can produce up to 200,000 seeds per plant (Weaver and Downs, 2003). Although research has identified a variety of control options for these species, particularly herbicide (e.g. Westra et al., 1992), the continued invasion of these species (largely due to herbicide resistance; Lu et al., 2007) highlights the need for better control techniques.

In the strip-seeding design, plots were exposed to different coverages of seeding, ranging from 0% of the plot area seeded (control) to 100% of plot area seeded. Five years after seeding, the experiment attempted to address if strip-seeding is useful for reducing the growth and reproduction of (1) field bindweed and (2) prickly lettuce. Specifically, we investigated how different levels of a strip-seeding treatment (i.e. percent of a given area that is seeded) might differentially affect invasive plant cover. The seeded species were predominantly bunchgrasses that are photosynthetically active for more months during the season and tend to reach peak biomass later in the season than most invasive annual grasses in the region. However, they overlap (in time) considerably with the two target invasives during periods of water scarcity, enhancing opportunities for competitive interactions. We expected target invasives in the seeded areas would demonstrate less growth and lower flower production than those found in the unseeded areas because seeded species and target invasives temporally overlap in growth and late season moisture acquisition, increasing the strength of competitive dynamics (Connell, 1983). We also expected target invasives in unseeded areas to respond unfavorably to higher seeding coverage in the long-term. This is because seed dispersal from seeded areas in higher coverage treatments would likely result in faster native species establishment in unseeded plots because of a greater seed pool, ultimately enhancing competitive pressure experienced by invasives (Seabloom et al., 2003).

## 2 Methods

### 2.1 Study area and experimental design

**Fig. 1:**
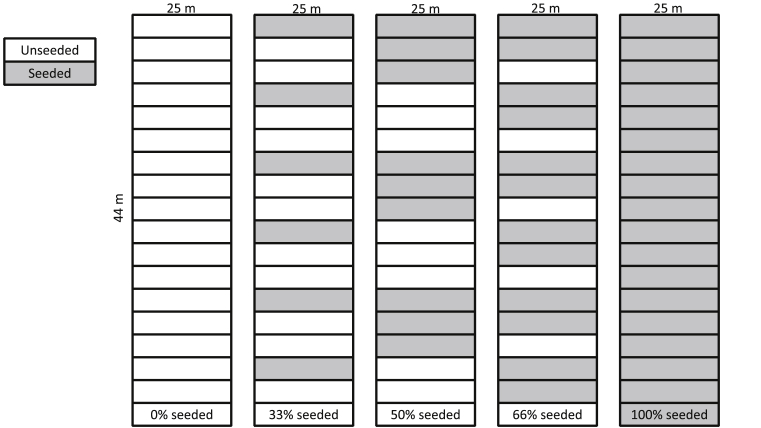
Design of the strip-seeding experiment.

The experiment was located in a grass field at the University of California, Davis (38.542865, -121.787842). The site has been historically used for grassland, pasture and agricultural experiments through the university. The soil is a Brentwood silty clay loam, and the area is characterized by a Mediterranean climate with 24.6 °C annual average temperature and 41 cm total annual precipitation that occurs mostly in the late fall and winter. The strip-seeding experiment was deployed in fall 2011 in order to address general weed dominance in an old-field. Two weeks after spraying with Roundup PowerMax (Glyphosate, 2.37 liters/ha) to kill existing plants at the site, native California grass mixes were drill seeded at 4 kg/ha into 44 m × 25 m plots in five different strip coverages (Table S1; Rayburn and Laca, 2013). The coverages included 0% seeded (no seeding of the plot area), 33% seeded (5 m unseeded strips separated by 2.4 m seeded strips), 50% seeded (7.2 m unseeded strips separated by 7.2 m seeded strips), 66% seeded (2.4 m unseeded strips separated by 4.8 m seeded strips), and 100% seeded (entire plot area seeded) (Fig. 1). The seeded mix included the perennials *Elymus glaucus* (blue wildrye), *Elymus multisetus* (big squirreltail), *Hordeum brachyantherum* (California barley), *Melica californica* (California oniongrass), *Poa secunda* (Sandberg's bluegrass), and *Stipa pulchra* (purple needlegrass), and the annual *Vulpia microstachys* (three weekfescue). Plots were randomly assigned within a block and blocks were replicated four times. Between seeding and data collection, plots and spaces between plots were mowed annually. Preliminary plant community surveys in 2015, four years after seeding, suggest that seeding treatments were effective in enhancing canopy cover (as determined by ocular surveys) of seeded species in seeded strips (average cover = 31%, dominated by *Stipa pulchra* and *Elymus glaucus*) compared to unseeded strips (average cover = 17%, dominated by *Elymus glaucus*), across treatments (J. Shaw, *unpublished data*). Prior to seeding (2011) and in the first year after seeding (2012), average cover of field bindweed and prickly lettuce across the experimental area was 0%, likely a result of initial herbicide application during site prep, and potentially in response to fairly high early establishment of some of the seeded species (J. Shaw, *unpublished data*). By 2016, the density of individuals was equal across treatment plots (approximately 3 individuals per meter squared in the unseeded strips and 1 individual per meter squared in the seeded strips).

### 2.2 Data collection

In June 2016 (at peak bloom), we measured the height, aboveground biomass and flower production of field bindweed and prickly lettuce individuals in seeded and unseeded strips across all plots. For field bindweed, we measured the length of the longest stem for height, and for both species, we included both open (fully developed) and unopened (buds) flowers in our counts for flower production. At the time of data collection, both target invasive species demonstrated approximately 25% cover in seeded and unseeded strips across treatments. There were no other late-season invasive plants present during June sampling. Five randomly chosen individual plants of each target invasive species were measured in one seeded and one unseeded strip in each plot (except for the 0% and 100% seeded plots, which only had one of each strip type available; n = 200 individuals for each species). After measuring height and flower number for each individual, aboveground plant biomass was harvested, placed in a drying oven at 55 °C for 48 hours, and weighed in the lab.

### 2.3 Analyses

In order to understand how seeding affects field bindweed and prickly lettuce, we used ANOVA models for an unbalanced design that included fixed factors of seeding treatment (unseeded strip and seeded strip), treatment coverage (0%, 33%, 50%, 66%, and 100% seeded areas of each plot), and interactions between the two, with replicate as the random factor. For each invasive species, we created three models to investigate experimental effects on target invasive plant height (bindweed error SS = 0.55; prickly lettuce error SS = 14.95), aboveground biomass (bindweed error SS = 3.46; prickly lettuce error SS = 32.36) and flower production (bindweed error SS = 557.1; prickly lettuce error SS = 145.24) for each species. In cases where independent variables contributed to differences in response variables, a TukeyHSD test was used to calculate differences within treatments. Assumptions of normality and homogeneity of variance were tested using boxplots and normal probability plots. All analyses were conducted in R version 3.2.0.

## 3 Results

### 3.1 Convolvulus arvensis

**Fig. 2:**
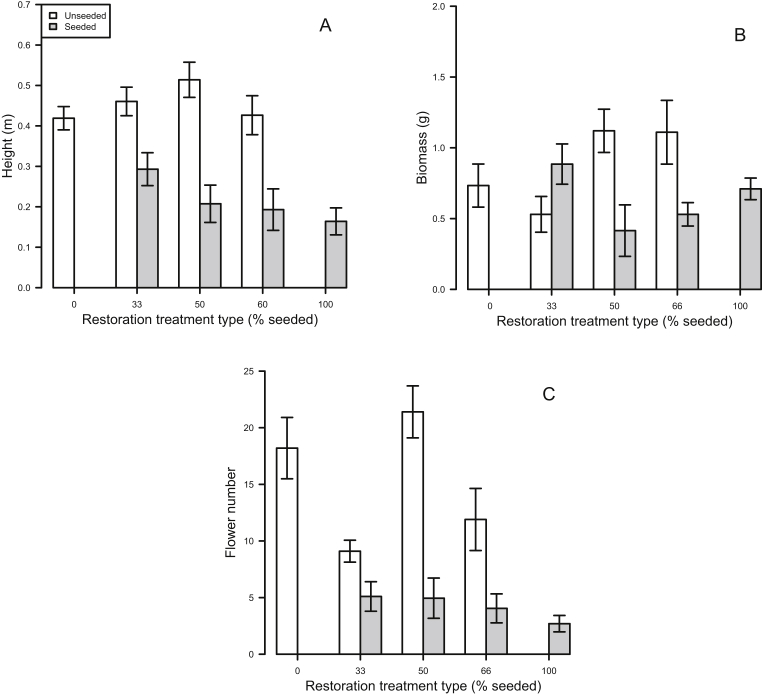
(A) Height (mean ± SD) of field bindweed across seeding coverages in unseeded (white) and seeded (gray) strips. (B) Aboveground biomass (mean ± SD) of field bindweed across seeding coverages in unseeded (white) and seeded (gray) strips. (C) Flower number (mean ± SD) of field bindweed across seeding coverages in unseeded (white) and seeded (gray) strips.

Field bindweed height was 18% shorter in seeded strips than unseeded strips (Fig. 2A; F_1, 132_ = 69.31, P < 0.001). Treatment coverage alone did not affect field bindweed height. Field bindweed biomass was 25% smaller in seeded strips than unseeded strips, across treatment levels (Fig. 2B; F_1, 132_ = 3.69, P = 0.05), but no main effect was found for treatment coverage. There was a significant interaction between the seeding treatment and treatment coverage (Fig. 2B; F_3, 159_ = 7.98, P = 0.005). The difference in biomass in seeded strips compared to unseeded strips appeared to be particularly large in plots with higher seeding coverage (50% and 66% seeded) but these differences were not significant in the posthoc test. Finally, field bindweed flower production was 71% lower in seeded strips than unseeded strips (Fig. 2C; F_1, 132_ = 109.24; P < 0.001). Overall, flower production was similar within seeded and unseeded strips across seeding levels.

### 3.2 Lactuca serriola

**Fig. 3:**
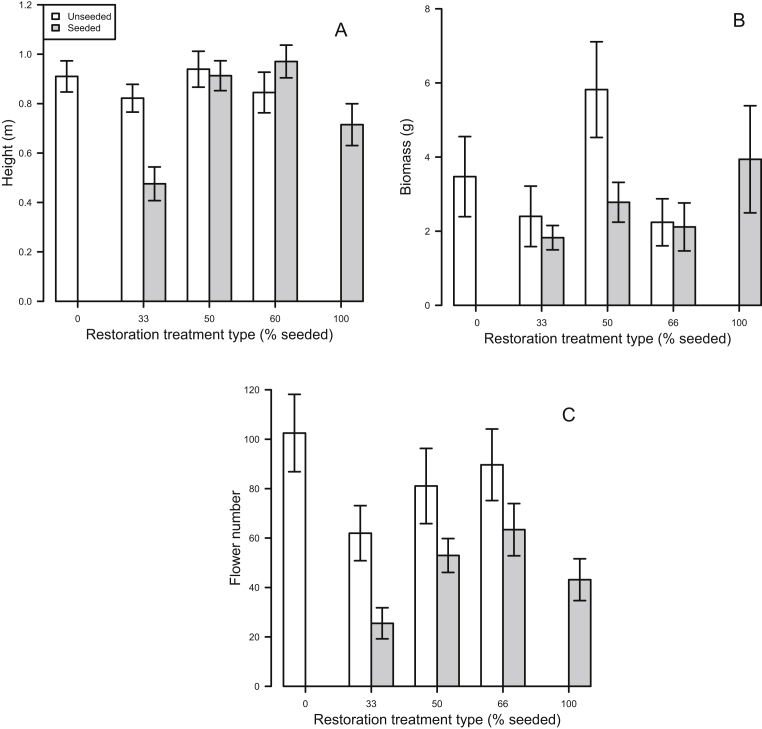
(A) Height (mean ± SD) of prickly lettuce across seeding coverages in unseeded (white) and seeded (gray) strips. (B) Aboveground biomass (mean ± SD) of prickly lettuce across seeding coverages in unseeded (white) and seeded (gray) strips. (C) Flower number (mean ± SD) of prickly lettuce across seeding coverages in unseeded (white) and seeded (gray) strips.

Plant height of prickly lettuce individuals in the seeded strips were 12% shorter than those in the unseeded strips (F_1, 132_ = 4.96, P = 0.001). This pattern was driven by an interaction between seeding and seeding coverage by treatment, where differences in height between seeded and unseeded strips in the 33% coverage plot was significant (Fig 3A; P = 0.05). No effect of any experimental treatments were found for prickly lettuce biomass (Fig. 3B). Prickly lettuce produced 45% fewer flowers per individual in the seeded strips than the unseeded strips (Fig. 3C; F_1, 132_ = 8.62, P = 0.003). We did not find a main effect of treatment coverage on flower production of prickly lettuce. Overall, height, biomass, and flower production were similar in unseeded strips, across seeding levels.

## 4 Discussion

The rapid modification of rangeland health in response to plant invasions highlights a critical need for the development of novel management strategies that enhance successful weed control. Seeding or planting desired species to address plant invasion can provide utility for management by facilitating competitive interactions (Berger, 1993) – one of the major factors driving plant invasion (Gioria and Osborne 2014). Seeding has shown promise in reducing invasive plant dominance when paired with conventional approaches (e.g. James et al., 2015). However, formal tests of strip-seeding strategies have largely been limited to agricultural systems. Here, we tested the utility of using strip-seeding to reduce the dominance of two prominent late season invasive plants: field bindweed, and prickly lettuce.

Reductions in plant height and flower production (for both target invasives), and aboveground biomass (for field bindweed) in seeded compared to unseeded strips support previous work that demonstrated the utility of seeding for weed control (Blumenthal et al., 2003). Competition from natives in the seeded strips are expected to be mechanism driving differences in target invasive characteristics because higher plant cover in these strips (mostly a result of high density of seeded species) compared to unseeded strips enhance overall competition for existing vegetation. There are several possible factors that could be mediating the effects of seeded species on late-season invasives. For example, changes in light availability has been shown to be a critical driver of invasion dynamics (e.g. Dyer and Rice, 1999; Siemann and Rogers, 2003). The presence of established perennial bunchgrasses likely reduce light availability at the soil surface, which can suppress photosynthetic stimulation of invasive plants (Blumenthal et al., 2003). Changes in soil moisture – a limiting resource in California grasslands – is another factor that can be responsible for changes in the relationship between native and invasive plants (Daehler, 2003). Because perennial bunchgrasses tend to allocate more energy into deep root development than invasives, established seeded species can appropriate limited late season soil moisture from deep in the soil profile, which potentially reduces invasive growth rate through desiccation stress (Holmes and Rice, 1996). Finally, native California grassland species have demonstrated the potential to enhance soil microbial communities that are antagonistic to or modify the growth patterns of invasive plants (Callaway et al., 2001, Callaway et al., 2003). The presence of established stands of seeded species could have modified components of the soil microbial community five years after seeding in a way that limited invasive plant growth and reproduction. It's likely that several mechanisms are operating simultaneously to mediate effects of native grass seeding on late-season invasives. In order to better understand the processes driving successful seeding approaches to control weeds, the relative contributions from aboveground competition and belowground factors to weed suppression need to be investigated further.

As expected, higher seeding coverages resulted in greater differences in biomass between seeded and unseeded strips for field bindweed. However, we found that prickly lettuce demonstrated a greater difference in height (Fig. 3A) between seeded and unseeded strips at a smaller seeding coverage. Generally, prickly lettuce has not been shown to compete strongly with surrounding vegetation (Carter and Prince, 1985), and early life history transitions are largely dependent on abiotic factors (Prince and Carter, 1985). Therefore, seeding coverages may not necessarily affect this weed because competition provided by seeded natives might not be a dominant driver of prickly lettuce establishment and further invasion. Alternatively, unseeded strips in the 33% seed coverage plots could have been characterized by a plant community that influenced the timing of prickly lettuce seed germination. Differences in germination timing influences survival in prickly lettuce (Marks and Prince, 1981), and might also play a role in biomass production. These mechanisms suggest that the way in which strip seeding provides utility for weed control can be species dependent.

Diversity in observed effects may also be due in part to unseeded patch size. The lowest seeding coverage treatment (33% of plot area seeded) is characterized by unseeded areas that are ‘thinner’ (e.g. two contiguous strips, 4.8 m width) than unseeded areas in the 50% coverage treatment (e.g. three contiguous strips, 7.2 m width; Fig. 1). As a result, unseeded strips in the 33% coverage treatment are likely exposed to higher seed rain (i.e. they are closer to seeded strips) than the 50% seeded plots. This highlights an important consideration for management design, where higher seeding rates do not necessarily result in an accompanying linear increase in weed control.

We did not find evidence that seeded strips provide invasion resistance to unseeded strips. This was unexpected as plant patch characteristics, such as patch size and proximity of neighboring patches, are known to affect factors that play a role in plant interactions (Magrach et al., 2014). It is possible that the established natives in the seeded patches have simply not yet dispersed and grown to a sufficient cover in the unseeded strips to negatively affect late-season invasives in these areas (e.g. Thomson et al., 2016). The consideration of plant traits in seeded species choice could provide managers with higher likelihood of successful invasion resistance in unseeded areas. For example, a species with high dispersal and rapid establishment could provide a more rapid colonization of unseeded areas from seeded areas.

## 5 Conclusions

Strip-seeding of native plants is particularly promising for integrated pest management strategies, which seek to combine techniques to provide long-term prevention of pests, for two reasons: (1) seeding of native species that phenologically overlap with invasives appears to be an effective way to reduce weed height, biomass and flower production; and (2) strip-seeding allows for strategic spatial targeting of efforts to minimize project-wide costs, which is a key consideration for native plant seedings. While the details of seeding selection and design might produce different management outcomes depending on target weed identity (e.g., functional traits and life history of the seeded and target species), this general approach of integrating ecological principles and economic considerations provides a complementary tool for inclusion in integrated pest management strategies.

## Declarations

### Author contribution statement

Amanda Dechen Silva: Performed the experiments; Wrote the paper.

Leslie M. Roche: Analyzed and interpreted the data; Wrote the paper.

Elise S. Gornish: Conceived and designed the experiments; Analyzed and interpreted the data; Contributed reagents, materials, analysis tools or data; Wrote the paper.

### Funding statement

This work was partially supported by USDA-NIFA, Rangeland Research Program Grant #CA-D-PLS-2119-CG to Emilio Laca. Amanda Dechen Silva was supported by the Universidade de São Paulo, Brazil.

### Competing interest statement

The authors declare no conflict of interest.

### Additional information

No additional information is available for this paper.

## Appendix A Supplementary data

Table S1 _spl_1_spl_ Table S1 _spl_1_spl_
