# Supplementary material for: The use of strip-seeding for management of two late-season invasive plants
Source: Heliyon. 2019 May 22;5(5):e01772. doi: 10.1016/j.heliyon.2019.e01772 (PMC6535581; doi:10.1016/j.heliyon.2019.e01772)
Supplement: Table S1 _spl_1_spl_ [file mmc1.docx]

**Table 1.** Seeding rate specifications of species used in strip-seeding experiment.

| **Name** | **Origin** | **Pure Live Seeds/bulk lb.** | **Bulk lb/ac** | **Percent cover in 2016** |
| --- | --- | --- | --- | --- |
| *Poa secunda* | Yolo county | 680,000 | 1.5 | 1.0 |
| *Vulpia microstachys* | Yolo County | 330,000 | 1.0 | 1.0 |
| *Elymus multisetus* | Yolo County | 52,000 | 3.0 | 1.0 |
| *Melica californica* | Yolo County | 238,000 | 2.5 | 2.2 |
| *Stipa pulchra* | Yolo County | 50,000 | 6.0 | 10.9 |
| *Elymus glaucus* | Yolo County | 118,000 | 2.0 | 9.2 |
| *Hordeum californicum* | Colusa County | 141,000 | 2.0 | 1.0 |
